# Direct Gaze Holds Attention, but Not in Individuals with Obsessive-Compulsive Disorder

**DOI:** 10.3390/brainsci12020288

**Published:** 2022-02-19

**Authors:** Mario Dalmaso, Lara Petri, Elisabetta Patron, Andrea Spoto, Michele Vicovaro

**Affiliations:** 1 Department of Developmental and Social Psychology, University of Padova, 35131 Padova, Italy; lara.petri@studenti.unipd.it; 2 Department of General Psychology, University of Padova, 35151 Padova, Italy; elisabetta.patron@unipd.it (E.P.); andrea.spoto@unipd.it (A.S.); michele.vicovaro@unipd.it (M.V.)

**Keywords:** eye contact, obsessive-compulsive disorder, social attention, social cognition

## Abstract

The attentional response to eye-gaze stimuli is still largely unexplored in individuals with obsessive-compulsive disorder (OCD). Here, we focused on an attentional phenomenon according to which a direct-gaze face can hold attention in a perceiver. Individuals with OCD and a group of matched healthy controls were asked to discriminate, through a speeded manual response, a peripheral target. Meanwhile, a task-irrelevant face displaying either direct gaze (in the eye-contact condition) or averted gaze (in the no-eye-contact condition) was also presented at the centre of the screen. Overall, the latencies were slower for faces with direct gaze than for faces with averted gaze; however, this difference was reliable in the healthy control group but not in the OCD group. This suggests the presence of an unusual attentional response to direct gaze in this clinical population.

## 1. Introduction

Obsessive-compulsive disorder (OCD) is a severe psychiatric disease characterised by the presence of repetitive and undesirable thoughts and behaviours, and it can also be considered a particularly restricting medical disorder [1]. According to the Diagnostic and Statistical Manual of Mental Disorders (DSM-5; American Psychiatric Association, 2013), OCD consists of two main components: obsessions, defined as persistent thoughts that are felt inappropriately and lead to anxiety (e.g., an exaggerated fear of contamination with harmful substances); and compulsions, which are repetitive mental acts (e.g., praying or silently repeating words) or actions (e.g., tidying up the house or body washing) put in place to counteract the torment caused by obsessions.

From a cognitive perspective, unusual attentional responses have often been reported in individuals with OCD. For example, some studies have documented the presence of attentional bias for stimuli related to OCD such as potential sources of contamination [2,3,4,5,6]. Other studies [7,8] described a reduced inhibition of return (i.e., a temporary inhibition of orienting towards spatial locations that were recently the focus of attention), likely reflecting a generalised inhibitory deficit that seems to characterize this clinical population [9,10].

Much less is known about potential alterations in OCD related to social attention, which is the attentional response to spatial cues from others [11]. Social attention is a core ability as it allows individuals to effectively navigate through social environments and establish meaningful social relationships [12]. According to the vast literature, social attention is largely guided by eye-gaze direction which provides a direct, unequivocal, and rapidly interpretable source of information about where another individual is attending [12,13,14,15]. Eye-gaze stimuli can lead to different but complementary social attentional phenomena [13]. For instance, on one hand, averted-gaze stimuli can elicit attentional shifts towards the same spatial location—i.e., the gaze-cueing effect [16,17,18]. On the other hand, direct-gaze stimuli, namely stimuli that establish *eye contact* with the observer, can hold attention more strongly than averted-gaze stimuli [19,20]. This *attention-holding effect* can be interpreted as complementary to the gaze-cueing effect, as it would help individuals to monitor potential approaching behaviours within social environments [14].

To the best of our knowledge, only one study investigated social attention in individuals with OCD [21]. In [21], a group of individuals with OCD and a matched control group of healthy individuals completed a behavioural task requiring the evaluation of spatial distance between two human avatars (see also [22]). These two avatars either looked at each other (i.e., they appeared to establish eye contact with each other) or not. The main results showed that both groups were influenced by the direction of the gaze; participants judged the distance between the two avatars to be smaller when the avatars gazed at each other rather than towards opposite directions. However, this difference (i.e., the perceived distance between the two avatars either gazing at each other or not) was less evident in individuals with OCD. The authors suggested that attentional response or sensitivity to the direction of the eye gaze of others may be compromised in individuals with OCD. This suggestion aligns with the broader difficulties in social functioning that have frequently been reported in this clinical population (for a recent review, see [23]). The results reported in [21] are intriguing and invite more efforts to explore whether such impairments can also include other related mechanisms of social attention.

In the present study, we further explored social attention in OCD by focusing on the attention-holding effect elicited by direct-gaze stimuli. An elegant manual task to reveal this effect was proposed by Senju and Hasegawa [24] then utilised in subsequent works [25,26] (for oculomotor evidence, see also [27,28]). In [24], participants were presented with a central face irrelevant to the task (the photograph of a woman’s face cut into an oval) with direct gaze (i.e., eye-contact condition), averted gaze, or closed eyes (i.e., no-eye-contact condition). After a stimulus onset asynchrony (SOA) of 500 or 1200 ms, a peripheral target appeared, and participants were asked to press a button as soon as they detected it. The main results showed that manual responses were slower when the face appeared with direct gaze compared to the other two conditions (i.e., the attention-holding effect). This was true at the 500 ms SOA but not at the 1200 ms SOA, suggesting that this phenomenon emerges early and decays quickly. In the present study, participants were presented with task-irrelevant faces that could display either direct gaze (i.e., eye-contact condition) or averted gaze (i.e., no-eye-contact condition), while a to-be-discriminated target appeared at a peripheral location. The performance of a group of individuals with OCD was compared to that of a matched group of healthy individuals. According to evidence showing reduced sensitivity to eye-gaze stimuli in individuals with OCD [21], we hypothesised that healthy controls would be slower to discriminate the target when it is presented in conjunction with a direct-gaze face rather than an averted-gaze face [24], and that this difference would be reduced in the OCD group.

## 2. Materials and Methods

### 2.1. Participants

Eighteen individuals (*mean age* = 32 years, *SD* = 10.611; *mean education* = 15 years, *SD* = 0.778; three women; two left-handed) diagnosed with OCD were recruited (the sample size was similar to other studies that explored social attention in clinical contexts, e.g., [29,30,31]). All individuals were treated at the Associazione Culturale Pandora located in Guamo (Lucca, Italy), a clinic specialising in the treatment of psychological and mental disorders. At the time of the test, the participants were receiving psychotherapy without pharmacological treatment, and none of them had other psychiatric disorders or a history of severe organic or neurologic pathology. Diagnoses were made by a board-certified attending research team of specialists using the International Classification of Diseases (ICD-10, World Health Organization, 1992). The control group consisted of 18 individuals (*mean age* = 32 years, *SD* = 10.605; *mean education* = 16 years, *SD* = 0.633; three women; two left-handed) without diagnoses of psychiatric illnesses or other organic or neurologic pathologies who were carefully selected from the local population to perfectly match the experimental group for age (*t*(34) < 0.001, *p* = 1, *d* < 0.001), education (*t*(34) = 0.391, *p* = 0.698, *d* = 0.130), handedness, and sex. All participants provided written informed consent. The study was approved by the Ethics Committee for Psychological Research at the University of Padova (protocol code: 3031; date of approval: 9 April 2019).

### 2.2. Stimuli, Apparatus, and Procedure

Our paradigm was similar to that used by Syrjämäki and Hietanen [26] in which avatar faces were employed. We chose avatars rather than real photographs (see [24]) because avatar faces provide the opportunity to present participants with well-controlled stimuli that are characterised by high ecological validity. The avatar faces—created using DAZ 3D software (ver. 4.10, Daz Productions, Inc., Salt Lake City, UT, USA; https://www.daz3d.com/, accessed on 16 February 2022)—were extracted from a set of stimuli employed in previous studies exploring social attention [32,33]. We used the face of a woman and the face of a man with the head oriented towards the observer (i.e., front view). Faces belonging to both sexes were used to increase ecological validity. For each face, one displayed direct gaze and one displayed averted gaze (closed-eye faces were not used to avoid perceptual confounds, as they are characterised by the absence of the sclera, iris, and pupil). A laptop running E-Prime handled the presentation of the stimuli. Participants were placed approximately 57 cm from a 15.6-inch monitor (1024 px × 768 px, 60 Hz). The background colour was set to grey.

**Figure 1:**
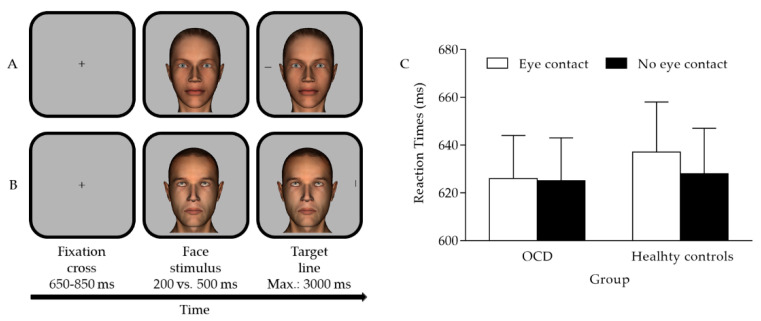
(**A**) depicts the female avatar face with direct gaze (i.e., eye-contact condition) and the horizontal target line appearing leftward; (**B**) depicts the male avatar face with averted gaze (i.e., no-eye-contact condition) and the vertical target line appearing rightward; (**C**) graphical representation of the eye-contact × group interaction in which the mean latencies observed for the eye-contact and the no-eye-contact conditions, within each group, are depicted (error bars are SEM).

Each trial started with a central fixation black cross (Arial font, 26-point size) presented for an interval chosen randomly within the 650–850 ms range. Then, a centrally placed face (10.7° width × 14.5° height) appeared for 200 or 500 ms (i.e., SOA). This face displayed either direct gaze (i.e., eye-contact condition) or averted gaze (i.e., no-eye-contact condition). Then, a target appeared 10.4° to the left or right of the centre of the screen. The target was a black line (1.3° width × 0.5° height) presented horizontally or vertically (see also Figure 1). Participants were asked to avoid eye movements and look at the centre of the screen for the whole duration of the trial. Moreover, they were instructed to discriminate the target orientation as quickly and accurately as possible by pressing one of two horizontally placed response keys. The association between the response key and the target orientation was counterbalanced among the participants. The trial ended when a response was provided or 3000 ms (timeout) passed, whichever came first. In case of incorrect or missed responses, visual feedback was provided for 800 ms (i.e., the words “error” or “missing response”, respectively). Finally, a blank screen appeared for an interval chosen randomly within the 800–1200 ms range, after which a new trial started. There was a practice block of 12 trials followed by an experimental block of 256 trials presented in random order (2 eye contact (eye contact vs. no eye contact) × 2 SOA (200 vs. 500 ms) × 2 face identity (man vs. woman) × 2 target orientation (horizontal vs. vertical) × 2 target position (leftward vs. rightward from the centre of the screen) × 8 repetitions). In the middle of the experimental block, a short break was provided.

## 3. Results

Data were handled and analysed as in [26]. Trials with missing responses (0.033% of the trials) or wrong responses (2.246% of the trials) were rare. These were discarded and not further analysed due to their low percentage. Correct trials with a latency smaller or greater than 2.5 SD of each participant’s mean (2.632% of the trials) were considered outliers and, in turn, discarded from subsequent analyses.

Mean latencies of the correct trials were analysed by mixed repeated measures ANOVA with eye contact (2: eye contact vs. no eye contact) and SOA (2: 200 vs. 500 ms) as within-participant factors and group (2: OCD vs. healthy control) as the between-participant factor (see also Table 1). A preliminary analysis with the Shapiro–Wilk normality test showed that, for all combinations of levels of the three factors, the data distribution was not significantly different from normal (*p* > 0.25). The main effect of eye contact was significant (*F*(1, 34) = 6.199, *p* = 0.018, *η*^2^*_p_* = 0.154) due to slower responses for the eye-contact condition (*M* = 631 ms, *SE* = 13.531) than for the no-eye-contact condition (*M* = 626 ms, *SE* = 12.834). The main effect of SOA was also significant (*F*(1, 34) = 135.851, *p* < 0.001, *η^2^_p_* = 0.800) due to slower responses at the 200 ms SOA (*M* = 648 ms, *SE* = 13.091) than at the 500 ms SOA (*M* = 610 ms, *SE* = 13.401), likely reflecting a foreperiod effect [34]. The main group effect was non-significant (*F*(1, 34) = 0.071, *p* = 0.791, *η^2^_p_* = 0.002). Importantly, the predicted eye contact × group interaction approached the canonical level of statistical significance (*F*(1, 34) = 3.869, *p* = 0.057, *η^2^_p_* = 0.102). No other significant results emerged (all *F* values < 2.947 and all *p*-values > 0.095), including for the eye contact × SOA × group interaction (*F*(1, 34) = 0.158, *p* = 0.694, *η^2^_p_* = 0.005). The absence of any interaction involving eye contact and SOA (all *F* values < 0.572 and all *p*-values > 0.455) was expected (see also [26]). The eye contact × group interaction was further analysed to test our a priori hypotheses. Hence, paired two-tailed *t*-tests comparing the eye-contact condition with the no-eye-contact condition were conducted for each group. As for the OCD group, the difference was non-significant (*t*(17) = 0.407, *p* = 0.689, *d* = 0.096), as the eye-contact condition led to similar response latencies (*M* = 626 ms, *SE* = 17.983) as the no-eye-contact condition (*M* = 625 ms, *SE* = 18.038). As for the healthy control group, the difference was significant (*t*(17) = 2.906, *p* = 0.010, *d* = 0.685), confirming that the eye-contact condition led to slower responses (*M* = 637 ms, *SE* = 20.661) than the no-eye-contact condition (*M* = 628 ms, *SE* = 18.777; see also Figure 1 panel C for a graphical representation of the eye contact × group interaction). Bayesian *t*-tests were also performed to determine which hypothesis (null vs. alternative) was better supported by the data. These tests indicated that, for the OCD group, the null hypothesis was almost four times more likely than the alternative hypothesis for the difference between the two conditions (i.e., eye contact vs. no eye contact; *BF_01_* = 3.817). In contrast, for the control group, the alternative hypothesis was more than five times more likely than the null hypothesis (*BF_10_* = 5.360) [35].

A direct test of our experimental hypothesis can also be obtained by comparing, across the two groups, the difference between the mean latencies for the eye-contact condition and the no-eye-contact condition. As we expected this difference to be larger in the control group than in the OCD group, a one-tailed independent sample *t*-test was performed. This showed that the difference was significantly larger in the control group (*M* = 9 ms, *SE* = 3.154) than in the OCD group (*M* = 1 ms, *SE* = 2.640; *t*(34) = 1.967, *p* = 0.029, *d* = 0.656). The Bayesian *t*-test showed that the alternative hypothesis was almost three times more likely than the null hypothesis (*BF*_10_ = 2.67).

## 4. Discussion

In the present study, we explored social attention in individuals with OCD by focusing on the attention-holding effect elicited by direct-gaze stimuli. A group of individuals with OCD and a matched group of healthy individuals were asked to discriminate a peripheral target. Meanwhile, an irrelevant central face with direct gaze (i.e., eye-contact condition) or averted gaze (i.e., no-eye-contact condition) was also presented. The main results showed that in healthy participants, latencies were slower in the eye-contact condition than in the no-eye-contact condition—thus replicating the main finding reported in [24]—whereas this difference was not reliable in individuals with OCD.

Decreased sensitivity to eye-gaze stimuli shown by individuals with OCD resembles the results from [21] which reported a weaker influence of eye-gaze stimuli in OCD during a perceptual task. More generally, this reduced sensitivity to eye-gaze stimuli seems to align with the broader literature showing some impairments of mechanisms that support social cognition in OCD. For example, there is evidence suggesting that those with OCD show reduced ability to understand the intentions and beliefs of others, i.e., the theory of mind, which can be involved in shaping social attention [36]. They may also show reduced sensitivity to facial stimuli such as impairments in recognising facial expressions and identities. However, the true nature of these deficits is still under debate, and research has produced mixed results [37,38,39,40,41,42]. From a more neuropsychological perspective, the existence of an altered attentional response to eye-gaze stimuli in OCD can find support at the neural level. According to several theoretical accounts and models [19,20,43,44,45], social attention abilities are supported by a relatively large and complex brain network. This includes the subcortical region of the amygdala which is activated to a greater extent by direct-gaze stimuli than by averted-gaze stimuli [46,47,48]. Reduced amygdala volume [49] and reduced activity for neutral faces [50] have been documented in individuals with OCD. Therefore, it can be hypothesised that the reduced effect of attention holding for direct gaze that we observed in individuals with OCD is associated with atypical functioning of the amygdala region.

As mentioned in the introduction, the task proposed in [24] has only been used in two studies that used manual responses [26,51]. However, these led to divergent results compared to the original study. In [25] in which a real individual (i.e., a confederate) either established or did not establish eye contact with the participant, faster responses were associated with the direct-gaze condition than with the averted-gaze condition. This may reflect the enhanced autonomic activation that is typically elicited by live faces with direct gaze [19,43] which, in turn, may cause enhanced target processing. The attention-holding effect for direct-gaze faces was replicated in [26] but only in a subsample of the responders (i.e., those who were primed with an induced condition of social inclusion). Our paradigm was heavily inspired by that of [26], but in our case, the latencies were overall longer for direct-gaze stimuli than for averted-gaze stimuli (i.e., the main effect of eye contact was significant), replicating the main finding of [24]. We believe that the main difference between our task and the task used in [26] is in the way that head stimuli were presented to the participants. In our task, heads were presented in front view, whereas in [26] they were presented 20° rotated on the vertical axis; this may have somehow weakened the perception of direct gaze (see also [11]). This speculative explanation (see also [26]) should be tested in specific studies.

One limitation of the present study is that it was impossible to collect standardised measures of clinical tests at the time of testing. This prevented the possibility of exploring any potential relationship between these clinical variables and the attentional phenomenon investigated in this study. Therefore, more research is necessary to capture the extent to which the unusual attentional response to direct gaze is associated with clinical measures of OCD, thus extending our preliminary data. Other avenues of future studies include the adoption of specific tasks to directly explore the discrimination of eye-gaze direction in OCD, as well as the adoption of more direct and ecological measures of attentional responses such as eye movements. During social interactions, we typically perform several eye movements to explore the social environment around us as well as to respond to social signals coming from others [52,53,54]. Studies on oculomotor measures have revealed new insights on the impact of eye contact on visual attention [32,55,56]. Therefore, the use of eye movements could also be beneficial for studying both face exploration dynamics and social attentional mechanisms in OCD. For example, a reviewer suggested that our results may reflect a tendency to avoid eye contact in individuals with OCD; this possibility can be effectively addressed using eye-tracking methodology.

## 5. Conclusions

To conclude, our study presents initial evidence supporting the notion that the attentional response to eye contact is compromised in individuals with OCD. This study extends previous evidence on social attention [21] and aligns with the general observation that social functioning is impaired in this clinical population [23].

## Figures and Tables

**Table 1:** Mean latencies (in ms) and SEM observed in both groups in all experimental conditions.

	200 ms SOA		500 ms SOA	
	Eye Contact	No Eye Contact	Eye Contact	No Eye Contact
OCD individuals	647 (18.423)	647 (18.463)	605 (17.961)	603 (18.077)
Healthy controls	652 (20.581)	645 (18.055)	622 (21.089)	611 (19.685)

## Data Availability

Data and stimuli associated with this study can be found on the OSF at the following link: https://doi.org/10.17605/OSF.IO/6N4TY.
